# Sensing the electrical activity of single ion channels with top-down silicon nanoribbons

**DOI:** 10.1088/2399-1984/aac737

**Published:** 2018-06-12

**Authors:** Weiwei Zhou, Luye Mu, Jinfeng Li, Mark Reed, Peter J Burke

**Affiliations:** 1 Department of Electrical Engineering and Computer Science, University of California, Irvine, CA, United States of America; 2 Department of Electrical Engineering; Department of Applied Physics, Yale University, New Haven, CT, United States of America

**Keywords:** ion channel, nanowire, electrophysiology

## Abstract

Using top-down fabricated silicon nanoribbons, we measure the opening and closing of ion channels alamethicin and gramicidin A. A capacitive model of the system is proposed to demonstrate that the geometric capacitance of the nanoribbon is charged by ion channel currents. The integration of top-down nanoribbons with electrophysiology holds promise for integration of electrically active living systems with artificial electronics.

## 1. Introduction

Ion channels are membrane-bound protein complexes and are important in a variety of biological processes, including neural function, cardiac function, pancreatic beta cell function, organelle energy production (chloroplasts) and consumption (mitochondria), to name a few. Of order 1% of all genes in the human genome code for ion channels [1]. 25% of drugs target ion channel function [2]. In spite of this, the basic technique to study ion channel function has not changed for over 30 years. Because of their exquisite charge sensitivity (with noise levels much less than a single electron or ion charge [3]), nano-electronic devices have the potential to revolutionize the way that electrophysiology measurements are performed. A critical first step in this direction is demonstration of sensing of currents from individual ion channels. We recently demonstrated this using bottom-up carbon-based nano-electronic platforms, including 1D (nanotubes) [4,5] and 2D (graphene) [6] allotropes of carbon. Although these are amenable to parallelization, there are additional challenges with new materials. On the other hand, silicon is a mature and stable technology with a pre-existing, vast, and global manufacturing base. We recently developed a top-down silicon nanoribbon technology that can enable high yield, sensitive devices that have been demonstrated in a variety of chemical and biological sensing contexts [7, 8]. Here we show sensing of single ion channel activity using silicon nanoribbons as the sense electrodes. The canonical ion channels alamethicin and gramicidin A (gA) were incorporated into supported lipid bilayers (SLBs) on top of the silicon nanoribbons, and a current was registered from the nanoribbon, though the ion channel, and into the solution. Each time a single, individual ion channel opens. This research lays the foundation for a silicon-based, top-down, economical, and massively parallel technology to probe ion channel electrophysiology.

## 2. Methods

### 2.1. Fabrication of silicon nanoribbon sensors

The process started with p-type 4’ ultra-thin silicon (70 *μ*m)-on-insulator wafer (SOITEC), with the active silicon layer thinned down to 40 nm through oxidation and buffered oxide etching. Alignment marks and backgate contacts were etched into the wafer, and the source, drain, and backgate were doped by BF_2_^+^ ion implantation at 10 keV with a dose of 2 × 10^15^ cm^−2^. Nanoribbon devices were defined in the active silicon layer using Cl_2_-based reactive ion etching (RIE). For Al_2_O_3_-passivated devices, a 20 nm thick conformal layer of AI_2_O_3_ was then deposited over the entire wafer using plasma-assisted atomic layer deposition (ALD) at Cornell NanoScale Science and Technology Facility, after which thermal annealing at 900 °C for 10 min was carried out to improve the quality of Al_2_O_3_ and performed dopant drive-in. The Al_2_O_3_ covering the source, drain, and backgate were removed using a combination of RIE and buffered oxide etching. Next, 200 nm of aluminum was deposited by electron beam evaporation and patterned by lift-off as contact metallization. To minimize leakage current between the devices and solution, a 2 *μ*m layer of SU-8 was deposited over the entire wafer at the end and used to generate a 20 *μ*m × 25 *μ*m window over the nanoribbons for sensing. SiO_2_-passivated devices were fabricated in the same manner, with the exception that instead of using ALD for dielectric deposition, 20 nm of SiO_2_ was thermally grown by dry oxidation at 1100 °C on the nanoribbons. The silicon nanoribbon is 1 or 2 *μ*m in width and 20 *μ*m in length. The whole fabrication process flow shown in figure S1 is available online at stacks.iop.org/NANOF/2/025008/mmedia. After fabrication, the chips were packaged in a dual in-line package with a polypropylene cup glued to the surface as a solution chamber with a diameter of ~4 mm. The exposed wirebonds are sealed by epoxy glue to protect them from electrolyte solution.

### 2.2. Preparation of lipid vesicle solution

A chloroform solution containing 4.25 mg 1,2-diheptadecanoyl-sn-glycero-3-phosphocholine (DPhPC) lipid was evaporated under a nitrogen stream for 2 h, forming a uniform lipid film on the bottom of a glass vial, and further dried in a desiccator under vacuum overnight. The dried lipid films were rehydrated in 5 ml 1 × PBS buffer to have a lipid concentration of ~ 1 mM, and subsequently sonicated for ~ 1 h until the solution appeared clear. The suspension was extruded at least 20 times through a mini-extruder with a 0.1 *μ*m pore size membrane (Avanti Polar Lipids). The lipid vesicle solution was stored at 4 °C overnight for later use.

### 2.3. Preparation of SLBs by the vesicle fusion method

A silicon nanoribbon chip was soaked in a hot deionized (DI) water bath (90 °C) for 1 h to generate a hydrophilic surface on silicon nanoribbons. After cooling down to room temperature, the chip was further stored in DI water overnight. DI water solution was exchanged to DPhPC lipid vesicle solution in the polypropylene cup on the chip surface by two micropipettes before vesicle fusion process. The chip was incubated in an oven for 2 h at 60 °C, followed by cooling down to room temperature. The unbound lipids were removed through rinsing with copious amounts of 1 × PBS buffer. The chip was stored at 4 °C overnight for further characterization.

### 2.4. Fluorescence imaging and fluorescence recovery after photobleaching (FRAP)

To obtain fluorescence images, DPhPC lipids were mixed with 1 mM fluorescent dyes Lissamine^™^ Rhodamine B 1,2-Dihexadecanoyl-sn-Glycero-3-Phosphoethanolamine, Triethylammonium Salt (LR-DHPE) at the molar ratio of 1000:1. No packaging was performed on silicon nanoribbon chips used for fluorescence imaging purpose. The chip was completely immersed in DPhPC vesicle solution in a sealed glass vial with a diameter of 12 mm during vesicle fusion process. The wet chip was taken out from the vial and gently washed with 1 × PBS buffer to remove unbound lipids. To prepare the solution reservoir, a PDMS well was mounted on a cover slide with the size slightly larger than the chip. For fluorescence imaging purpose, the wet chip was placed upside down into the PDMS well. More 1 × PBS buffer was immediately injected into the PDMS well to prevent the chip from drying. FRAP experiments were performed to study lipid fluidity by a Zeiss LSM 780 confocal laser microscope with an excitation laser at 561 nm. The 561 nm laser was set at 100% intensity to bleach a spot in the lipid membrane. The fluorescence recovery was recorded with a 50× objective lens with 5 s intervals between images at a reduced laser power (<4 mW). The diffusion coefficients of SLBs were calculated by measuring percentage recovery of fluorescent intensity of the bleached spot in time lapse.

### 2.5. Electrical measurement and data acquisition of ion channel recording

The voltage-gated ion channel alamethicin was incorporated into preformed SLBs by adding its ethanolic stock solution onto the polypropylene cup mounted on chip surface resulting in a typical concentration of 2 *μ*M. To reconstitute gA into SLBs, 0.2 mM gA was added into DPhPC chloroform solution at the molar ratio of 100:1 (final concentration of 2 *μ*M) before the solvent evaporation process, and then mixed for two hours at room temperature followed by solvent evaporation. 0.5 M KCl in 1 × PBS solution was used for recording. Two micropipettes were used for liquid exchange. Ion channel recordings were typically performed at an applied potential of +100 mV using a patch clamp amplifier system as previously described [4]. Data were low pass filtered at 5 kHz using the 4-pole Bessel filter built into the Axopatch 200B and sampled at 10 kHz. Data collection was done by electrophysiology software (pClamp10) and plotted using Igor Pro.

## 3. Results and discussion

### 3.1. Surface functionalization of top-down silicon nanoribbon with SLB

**Figure 1.**
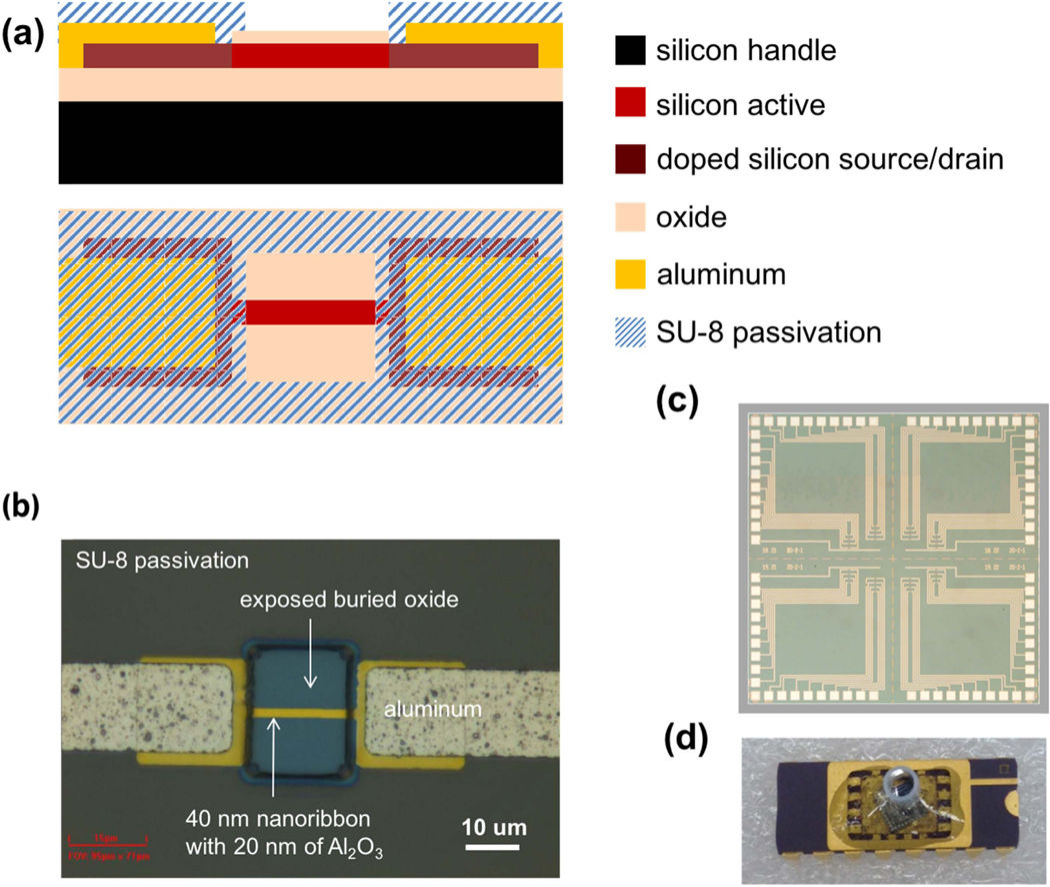
(a) Cross section and top view layer schematics of silicon nanoribbon device, as well as optical micrograph at various magnifications, including (b) single device, (c) single chip, and (d) packaged chip.

To begin, we fabricated top-down silicon nanoribbon devices with windows exposing only the nanoribbon/oxide surface to solution (figure 1). Figure 1 (b) shows an optical image of a 2 *μ*m wide and 20 *μ*m long silicon nanoribbon. *p*-type Al_2_O_3_-passivated and SiO_2_-passivated devices were fabricated similarly to previously reported recipes [8, 9]. The fabrication process was carefully controlled such that the final thicknesses of the silicon nanoribbon and dielectric are 40 nm and 20 nm, respectively. The fabrication process is demonstrated in figure S1. After fabrication, the chips were each packaged in a dual in-line package with a polypropylene cup glued to the surface as solution chamber except for the chips used for fluorescence experiments.

**Figure 2.**
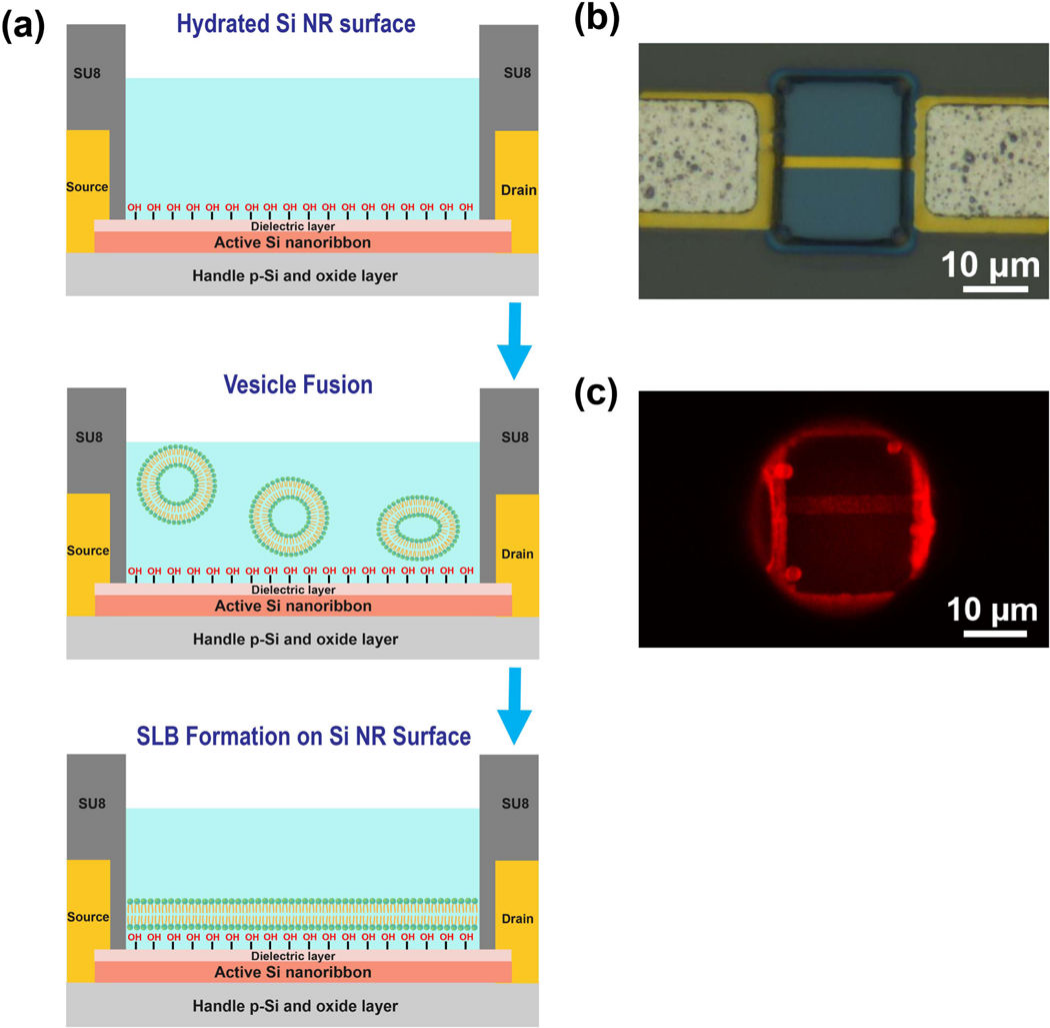
(a) Schematic illustration of the formation of SLBs on silicon nanoribbon. (b) Bright field image and (c) fluorescence image of SLBs on single silicon nanoribbon device.

Following device fabrication, lipid bilayer functionalization of the nanoribbons was carried out using the vesicle fusion method (figure 2). The bilayers adhered to the charged, hydrophilic surface of the oxide via physisorption. The recipe was similar to our recent work [4, 6]. Fluorescence (figure 2(c)) and FRAP (figure S5) show that the lipid bilayer formation was uniform and allowed diffusion of the lipids readily in plane. We used unpackaged chips for fluorescence imaging because the glue and the polypropylene cup on the packaged chip prevent the high magnification objective lens from approaching the chip surface during the imaging process. As described in the Methods section, the only difference between using the unpackaged and packaged chips in the fluorescence experiment is that we performed vesicle fusion processes for unpackaged chips in a ~ 12 mm diameter glass vial but for packaged chips performed in a ~4 mm diameter polypropylene cup. The polypropylene cup slightly restrained the electrolyte solution in comparison to the glass vial, however it still can be considered as an open environment for nanoribbon device when compared to the micro-size SU-8 sensing window. It is completely different with the scenario of using a microfluidic channel to create a micro/nano liter space. Therefore, the formation of SLBs on the nanoribbon should not be significantly affected by using packaged or unpackaged chips.

**Figure 3.**
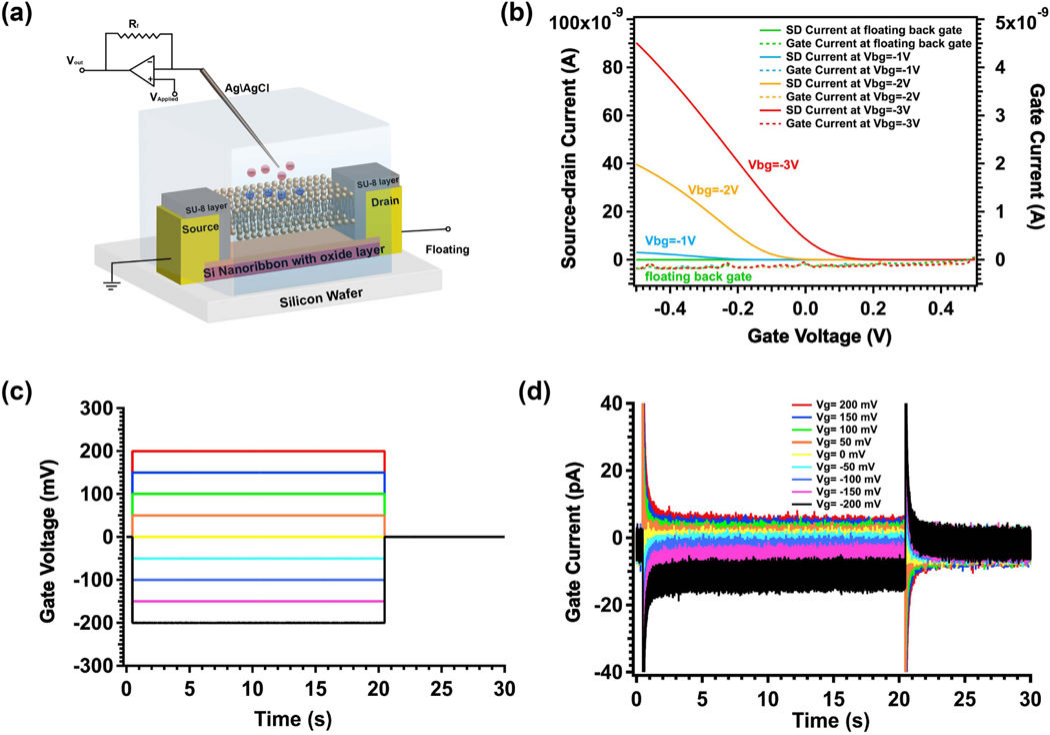
Electrical properties after functionalization (without ion channels), demonstrating a low-leakage, high quality lipid bilayer seal over the silicon nanoribbon device. (a) Schematic illustration of electrical seal test of a lipid bilayer functionalized nanoribbon. (b) Depletion curve showing back gate tuning of the threshold voltage before vesicle fusion. (c) Waveforms of gate voltage to test leakage after lipid bilayer formation. (d) Leakage currents at different gate voltages through lipid bilayer showing an excellent seal (>10 Gohm) after lipid bilayer formation.

Due to the multiple patterned layers used to fabricate the nanoribbons, the resulting chip surface is not entirely planar. The step height resulting from the nanoribbon is ~60 nm, however, the SU-8 via introduces a step height of 2 *μ*m in the vicinity of the nanoribbon. This 3D relief may affect the quality of the deposited bilayer over the nanoribbon. We carefully screened for devices that had a GΩ electrical seal, as determined by the current from the nanoribbon, through the bilayer, to the solution, in the absence of ion channels, to ensure there were no pinholes or unsealed regions of the lipid bilayer or insulator (figure 3(d)).

Before and after lipid bilayer deposition, the electrolyte served as the gate to turn the nanoribbon on and off. The charged lipid bilayer shifted the threshold voltage to be more negative after deposition (figure S2). This trend was reversible: after washing with sodium dodecyl sulfate, the electrical properties of the silicon nanoribbon were mostly recovered (figures S2(c), (f)), including the pH and electrolyte concentration dependence. A similar threshold voltage shift was also observed for carbon nanotubes functionalized with lipid bilayers [10], although the sign of the threshold voltage shift was not explained. In order to confirm our interpretation of the voltage shift, we performed layer by layer polyelectrolyte deposition (figures S3 and S4). The data clearly show the effect of a charged polymer on the threshold voltage of the nanoribbons. Although the lipid bilayer threshold voltage shift has not been studied before this work, similar polyelectrolyte results were published on silicon nanowires/nanoribbons in [9,11].

### 3.2. Electrical tests of silicon nanoribbon before and after SLB functionalization

For the ion channel recording from our devices, we used a patch clamp amplifier that has an operational amplifier with negative feedback that automatically maintains a constant applied potential. A two-electrode system was used for ion channel activity recording. A silver/silver chloride (Ag/AgCl) wire electrode was inserted into buffer solution over the cis chamber of the device. The voltage was applied to the chamber through Ag/AgCl electrode connected to the headstage of patch clamp system. The reference electrode is the silicon nanoribbon, which was contacted with ohmic contacts in turn connected to wire bond pads and a DIP chip. All the current measurements using patch clamp system were carried out inside a Faraday cage to shield the device from external electric fields. Potassium chloride (500 mM) was used as the measurement electrolyte.

For an unfunctionalized nanoribbon, electrolyte gate voltages of ~ 1–10 V are possible. However, the introduction of the lipid bilayer added an additional constraint on the gate voltage. Since a lipid bilayer can sustain only ~0.2 V, it was not possible to bias the electrolyte much higher than this without damage to the bilayer. Although with additional effort it should be possible to pre-engineer the threshold voltage so that after bilayer deposition the nanoribbon was on at around 0 V, it is not possible with our existing process to precisely control the threshold voltage.

In order to address this issue, we used two approaches: first, we used Al_2_O_2_ instead of SiO_2_ as the oxide, as it was more likely in our process to result in the appropriate threshold voltage. Second, once fabricated, we used the substrate as a second (back) gate to fine tune the threshold voltage *after* bilayer deposition to enable the nanoribbon to be in the ‘on’ state at electrolyte voltages between −0.2 and +0.2 V (figures 3(b) and S2(g), (h)). The nanoribbon just needs to have a much lower resistance than the ion channel. Both of these approaches were successful. Once suitably functionalized with the appropriate threshold voltages, the devices displayed very little gate leakage current (figures 3(c), (d)). There are no redox active species in our chemistry, so we expect no redox currents at the oxide-solution interface. In addition, the oxide itself is expected to be insulating if the fabrication is performed properly and there are no microscopic pinholes.

### 3.3. Detection of ion channel activities by SLB functionalized silicon nanoribbon device

After establishing a method for lipid bilayer functionalization, we aimed to examine the current directly through ion channels using the silicon nanoribbons as electrodes. As in our previous work with carbon-based nano-electrodes, we chose gA and alamethicin as the demonstration ion channels. In suspended lipid bilayers, gA has an ohmic *I–V* curve with one open state, which opens for about 0.1s when two gramicidin monomers dimerize [12]. The open current is typically 10 pA at 100 mV. Alamethicin has a higher open state current (~100 pA), although it has multiple open states.

To reconstitute gA in SLBs, gA in ethanolic stock was premixed with DPhPC chloroform solution before the solvent evaporation process, while alamethicin was inserted to preformed SLB by directly adding to the chamber from their ethanolic stock.

**Figure 4.**
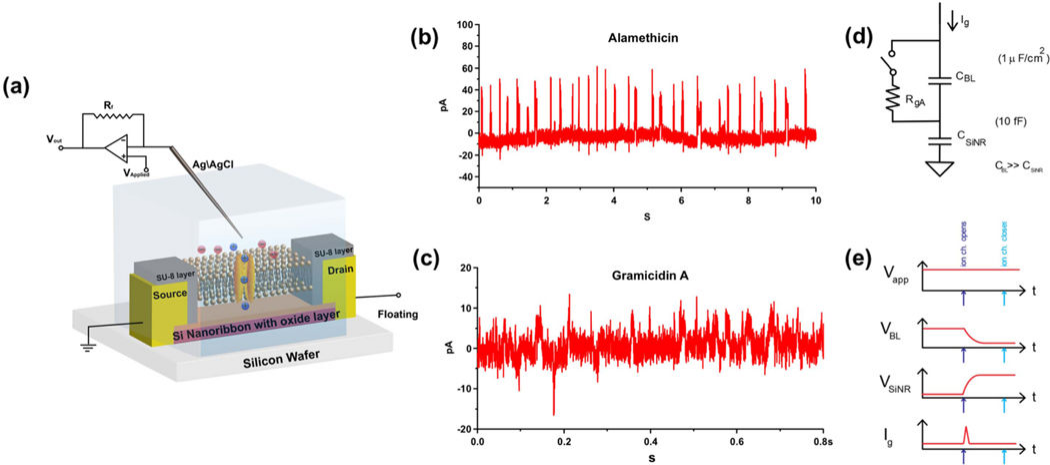
Current versus time from nanoribbon, though lipid bilayer, into solution, for alamethicin or gramicidin A incorporated into the bilayer. (a) Schematic illustration of a general strategy of using 1D silicon nanoribbon to detect ion current through ion channel pores. (b) Current trace for ion channel activities of alamethicin. (c) Current trace for ion channel activities of gA. Each spike is the opening of a single ion channel. V_applied_ = 100 mV. (d) Proposed circuit model showing the ion channel and relevant silicon nanoribbon and suspended lipid bilayer capacitances. (e) Dynamic model showing how charge builds up on the nanoribbon, quickly taking a large fraction of the applied voltage so no voltage is across the bilayer, shutting off the ion channel current, as observed experimentally.

We then used the setup of figure 4(a) to measure ion channel activity, figures 4(b) and (c) show current versus time from nanoribbon for alamethicin or gA, respectively. The measurements with alamethicin resulted in current spikes of an amplitude of ~50 pA at *V*_applied_ = 100 mV, with an average open time of 20 ms (figure 4(b)). For the gA measurements, we observed current spikes of order 10 pA, also with an average open time of 20 ms (figure 4(c)). The current steps are consistent with both alamethicin and gA behavior in suspended lipid bilayers. Thus, the amplitude of the steps, together with the fact the chemical recipe was virtually identical to our recent work with carbon nanoelectrodes [4,6], gives us confidence that the spikes are from individual ion channels opening and closing, demonstrating the first ever integration of silicon nanoribbons with individual ion channels. One curiosity is that the average spike width is shorter than that observed in traditional suspended lipid bilayers [12].

**Figure 5.**
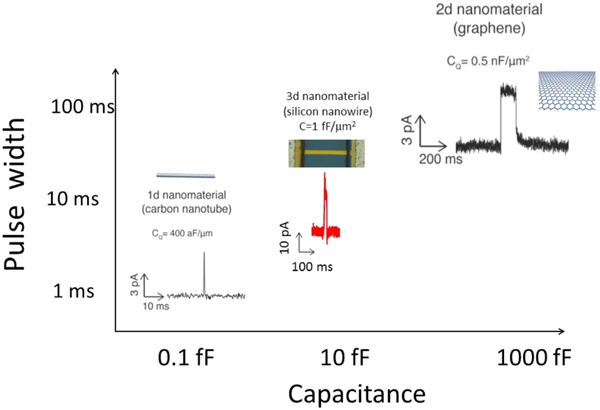
The pulse width is observed to scale with the nano-electrode capacitance across multiple technology platforms of differing materials composition: carbon and silicon and quantum dimensionality: 1D, 2D, 3D.

In order to better understand the timing of the current waveforms, in particular the spike width, we need to turn to an equivalent circuit model of our system (figures 4(d), (e)). We base our interpretation heavily on the recent interpretation of our carbon experiments [4, 6], where the ion channel current was found to capacitively charge the electrode. The quantum capacitance of 1d nanotube is about 6 orders of magnitude smaller than the quantum capacitance of 2d graphene (400 aF *μ*m^−1^ versus 0.5 nF *μ*m^−2^). In the case of a nanotube, the capacitance is ~ 1fF (figure 5) when a several or tens of pA current from a single ion channel charged it up to 100 mV in a few ms, effectively blocking additional ion channel current. In contrast, with graphene, the large area capacitance (figure 5) is ~mF for a mm^2^ area used that it never became charged before the ion channel naturally turned off (i.e. by physical dissociation of dimers in the case of gA or channel closing in the case of alamethicin).

We next turn to an estimate of the nanoribbon capacitance. We can estimate the total capacitance (*C*_*total*_) by considering the series capacitance of the solution double layer, dielectric, and the silicon channel. The double layer capacitance is 2 orders of magnitude larger than the nanoribbon capacitances in this case because of the large relative permittivity of solution and the small Debye length at high ionic strengths, and can therefore be neglected from our calculations. The detailed calculations for the capacitance of solution double layer, dielectric layer, and the silicon channel are shown in supporting information. The dielectric capacitance can be calculated using the relative permittivity (*ε*_*r,ox*_) of SiO_2_ and Al_2_O_3_ respectively, and an oxide thickness of 20 nm (*t*_*ox*_). The silicon capacitance can be estimated by assuming full depletion through the thickness of the nanoribbon, thus using 40 nm as the thickness (*t*_*si*_).

(1) $$C_{total}\approx {\left(\frac{t_{ox}}{\varepsilon_{0}\varepsilon_{r,ox}}+\frac{t_{Si}}{\varepsilon_{0}\varepsilon_{r,Si}}\right)}^{-1}$$

Here *ε*_0_ is the permittivity of free space which is 8.85 × 10^−12^ F m^−1^, and *ε*_*r,si*_ is the relative permittivity of silicon which is 11.7. Using equation (1), we can estimate the total capacitance to be approximately 1.6 fF *μ*m^−2^ and 1.0 fF *μ*m^−2^ for Al_2_O_3_ (*ε*_*r,ox*_ *=* 9) and SiO_2_ (*ε*_*r,ox*_ *=* 3.9) devices, respectively. For the nanoribbon device used in figure 4 with the dimension of 20 *μ*m × 1 *μ*m, the capacitance estimation yields 20–30 fF.

We now discuss how this fits into our circuit model (figure 4(d)). In our previous works, we developed a circuit model which demonstrated that the ion channel currents charged the capacitance of the nanotube/graphene system [4, 6]. In all our experiments (including this work), the total voltage drop is distributed across two capacitances in series: the lipid bilayer capacitance and the nano-electrode capacitance. The lipid bilayer capacitance is ~ 10 fF m^−2^ [4, 6]. This voltage is clamped by the voltage source in the circuit, typically at 0.1 V. When the ion channel opens, a current flow (of order 10 pA) through the ion channel and charges the nano-electrode capacitance. If this capacitance is an order of magnitude smaller than the lipid bilayer capacitance (as in the case of the carbon nanotube), it quickly charges up, so that the total voltage drop is dominantly across the nano-electrode capacitance, and not the bilayer. Thus the bilayer voltage becomes small, causing the current through the ion channel to drop back to zero. The exact time it takes depends on the capacitance of the nano-electrode system. In the opposite case, when the capacitance of the nano-electrode is an order of magnitude higher (for example our graphene experiments), the voltage across the bilayer remains a constant and the ion channel current does not change until the ion channel naturally closes. The silicon nanoribbon case should be somewhere between the graphene and nanotube.

The silicon nanoribbon experiments are firmly in the limit of small nano-electrode capacitance in comparison to the lipid bilayer capacitance, and therefore we expect the same phenomenon as the nanotube experiments. However, as the silicon nanoribbon has larger capacitance than the carbon nanotube (20–30 fF versus ~ 1 fF), we expect a concomitantly longer open time of the spike width. Indeed, this is exactly what we observe: for the nanotube, the spike width is ~ 1 ms, whereas for the silicon nanoribbon, the spike width is 20 ms. This interpretation demonstrates a consistent set of data across multiple technology platforms (of differing materials composition: carbon and silicon and quantum dimensionality: 1D, 2D, 3D) (figure 5).

## 4. Conclusions

In conclusion, we have shown integration with silicon nanoribbon electronics and single ion channel current measurements. This work is inherently scalable and can readily be applied to any ion channel of interest, of which there are thousands.

## Supplementary Material

1
