# Supplementary material for: Sensing the electrical activity of single ion channels with top-down silicon nanoribbons
Source: Nano Futures. Author manuscript; Available in PMC 2019 Jun 1. (PMC6390970; doi:10.1088/2399-1984/aac737)
Supplement: 1 [file NIHMS1013226-supplement-1.pdf]

Supporting information

Sensing the electrical activity of single ion channels with top-down silicon nanoribbons

Weiwei Zhou,[1] Luye Mu[2], Jinfeng Li[1], Mark Reed[2], Peter J. Burke[1]

[1]Department of Electrical Engineering and Computer Science, University of California, Irvine, CA, USA;
[2]Department of Electrical Engineering; Department of Applied Physics, Yale University, New Haven, CT, USA

Silicon nanoribbon device fabrication flow

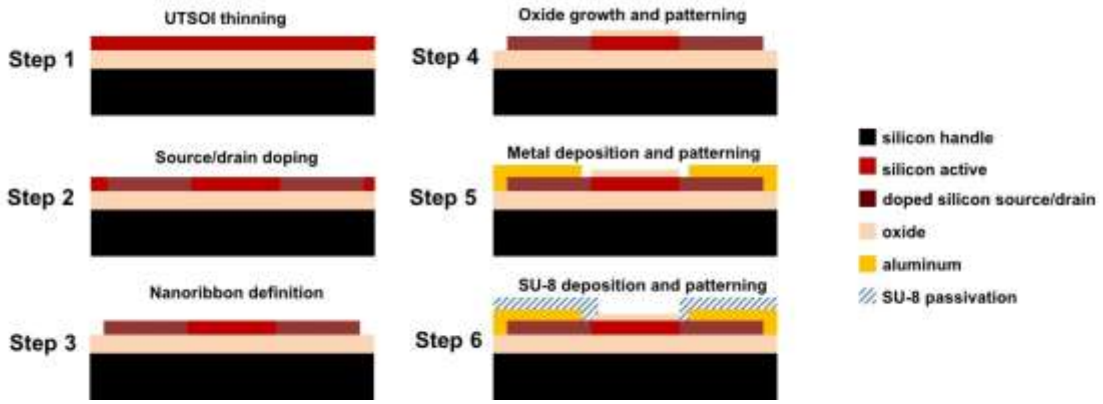

Figure S1. Demonstration cartoon of the fabrication flow of a silicon nanoribbon device.

Threshold voltage before and after lipid bilayer deposition

The depletion curves show significant changes before (figure S2(a, d)) and after lipid bilayer deposition (figure S2(c, f)). The silicon nanoribbon device with the threshold voltage of ~ 0 V could not be turned "on" within the safe gate voltage window of ± 0.2 V after the SLB formed. After 10% SDS washing, the electrical properties of the silicon nanoribbon were recovered. More devices with more positive threshold voltages have been tested and shown significant decreases of source-drain currents after lipid bilayer deposition. A representative device was

shown in figure S2(g, h). Therefore, we believe that there is a negative shift of the threshold voltage after the SLBs formed. A DPhPC lipid contains one positive charge and one negative charge, however, the positive charge at the head group is closer to silicon nanoribbon surface when the SLBs form, thus possibly resulting in the negative shift of the threshold voltage. A layer-by-layer polyelectrolyte deposition experiment (figure S3 and S4) was performed to obviously show this trend.

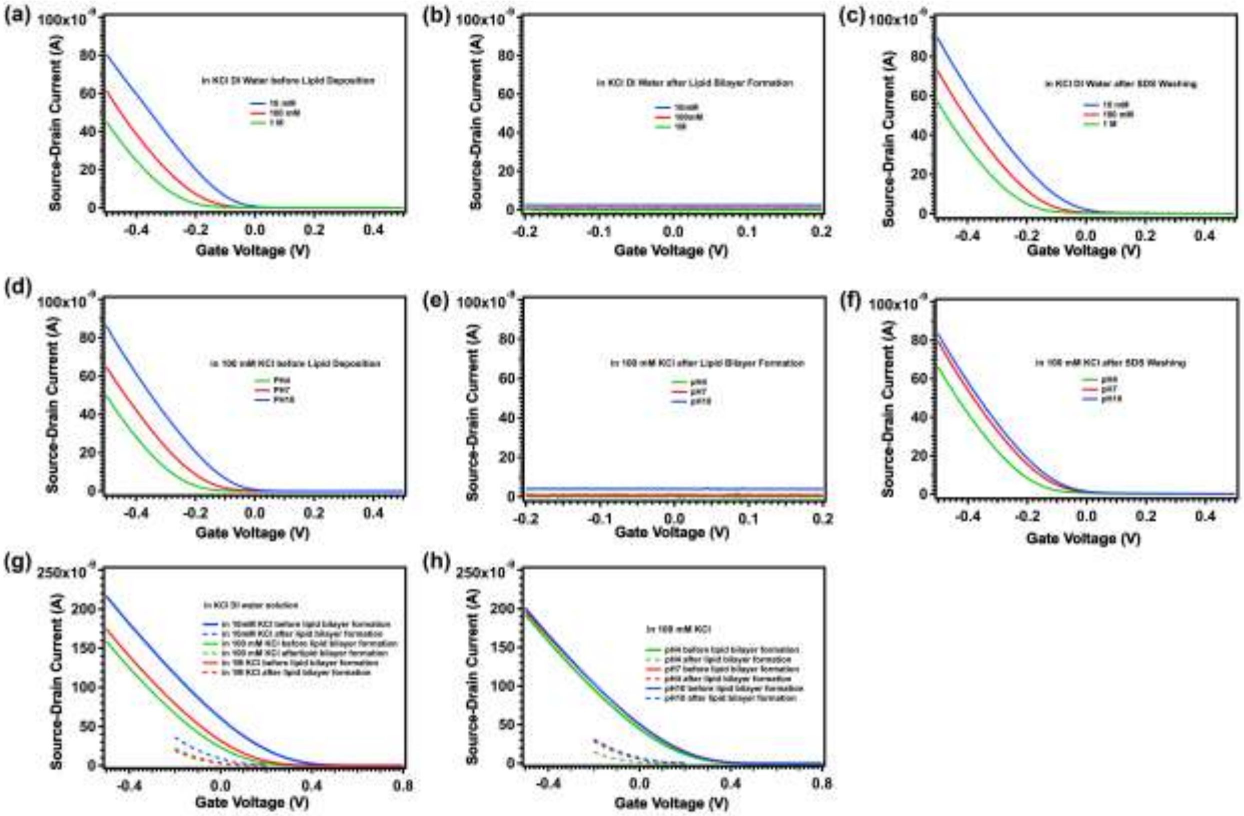

Figure S2. Depletion curves of a silicon nanoribbon device with the threshold voltage of ~ 0 V before (a, d) and after lipid bilayer deposition (b, e) and after lipid bilayer removal by SDS washing (c, f) in KCl solutions with varied concentrations and pH Values. Another silicon nanoribbon device with the threshold voltage of ~ 0.5 V was represented in (g, h) to show the negative shift of threshold voltage.

Layer by layer polyelectrolyte deposition:

In order to confirm our interpretation of the voltage shift, we performed layer by layer polyelectrolyte deposition. PSS and PDDA were dissolved in MES buffer (10 mM, 50 mM

NaCl, pH=6.0) at a concentration of 1 µg/mL. Similar results were reported by us on graphene in ref. [13] and by another group on silicon in ref. [11].

The figures below show the change in the surface potential (calculated as the change in drain current normalized by the device transconductance) upon sequential additions of charged polymers PSS and PDDA.

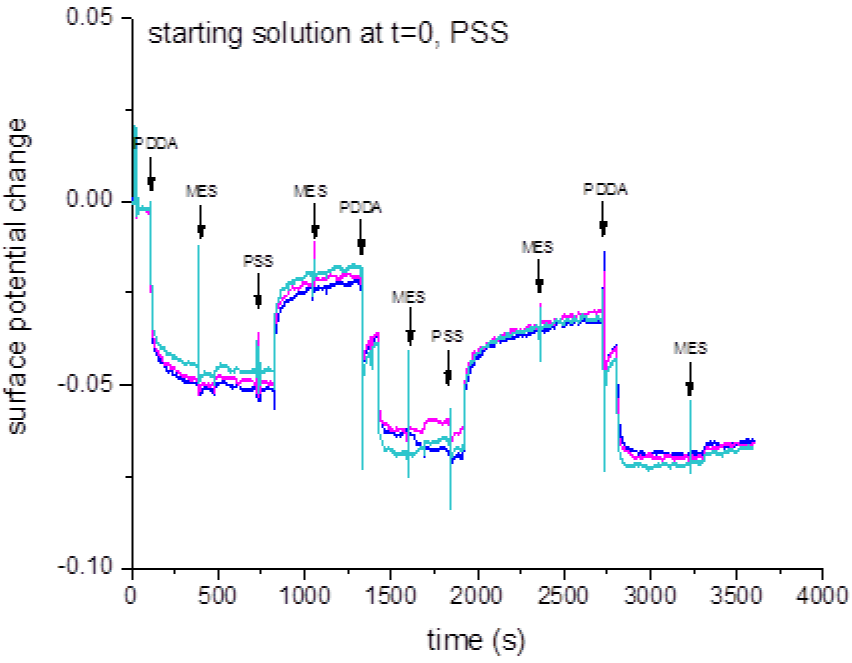

Figure S3: Surface potential vs. time of layer-by-layer deposition.

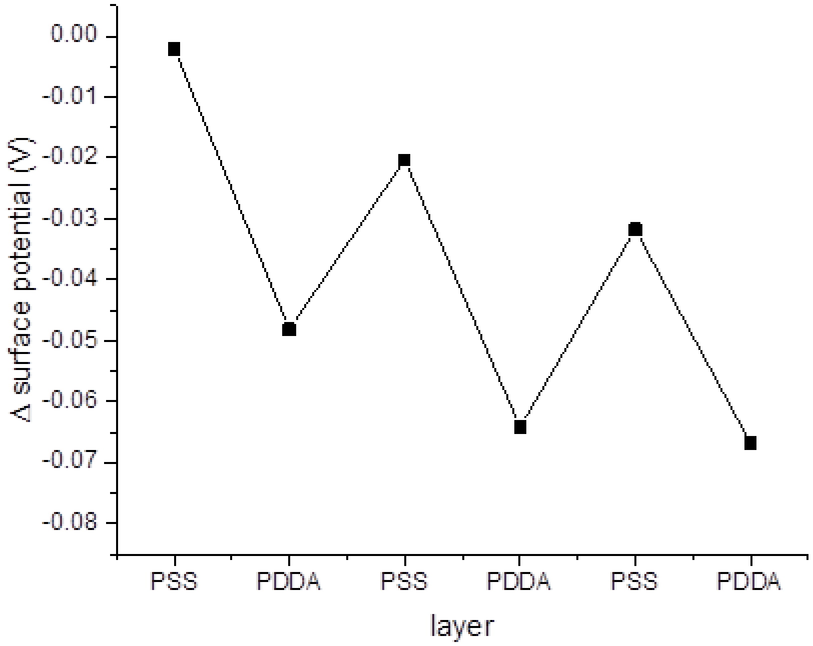

Figure S4: Surface potential upon sequential addition of PSS, PDDA.

Fluorescence imaging and fluorescence recovery after photobleaching (FRAP)

The FRAP analysis was performed to evaluate the lateral diffusion of SLB on silicon nanoribbons. The diffusion coefficient, D, can be determined by the equation: $D = 0.224\ r^2/t_{1/2}$, in which r is the radius of the photobleached area, and $t_{1/2}$ is the time required to achieve 50% fluorescence intensity recovery. Silicon nanoribbon arrays were used in this study instead of single nanoribbon devices. A representative experiment is shown below. Fluorescence images of a sample spot with a diameter of about 29 µm were recorded before (figure S5(a)), immediately after (figure S5(b)), and 276 seconds (figure S5(c)) after photobleaching. According to the FRAP recovery curve shown below, the diffusion coefficient D of ~0.71 $\mu m^2\ s^{-1}$ were calculated for LR-DHPE in the SLB with the mobile fraction F of ~92%.

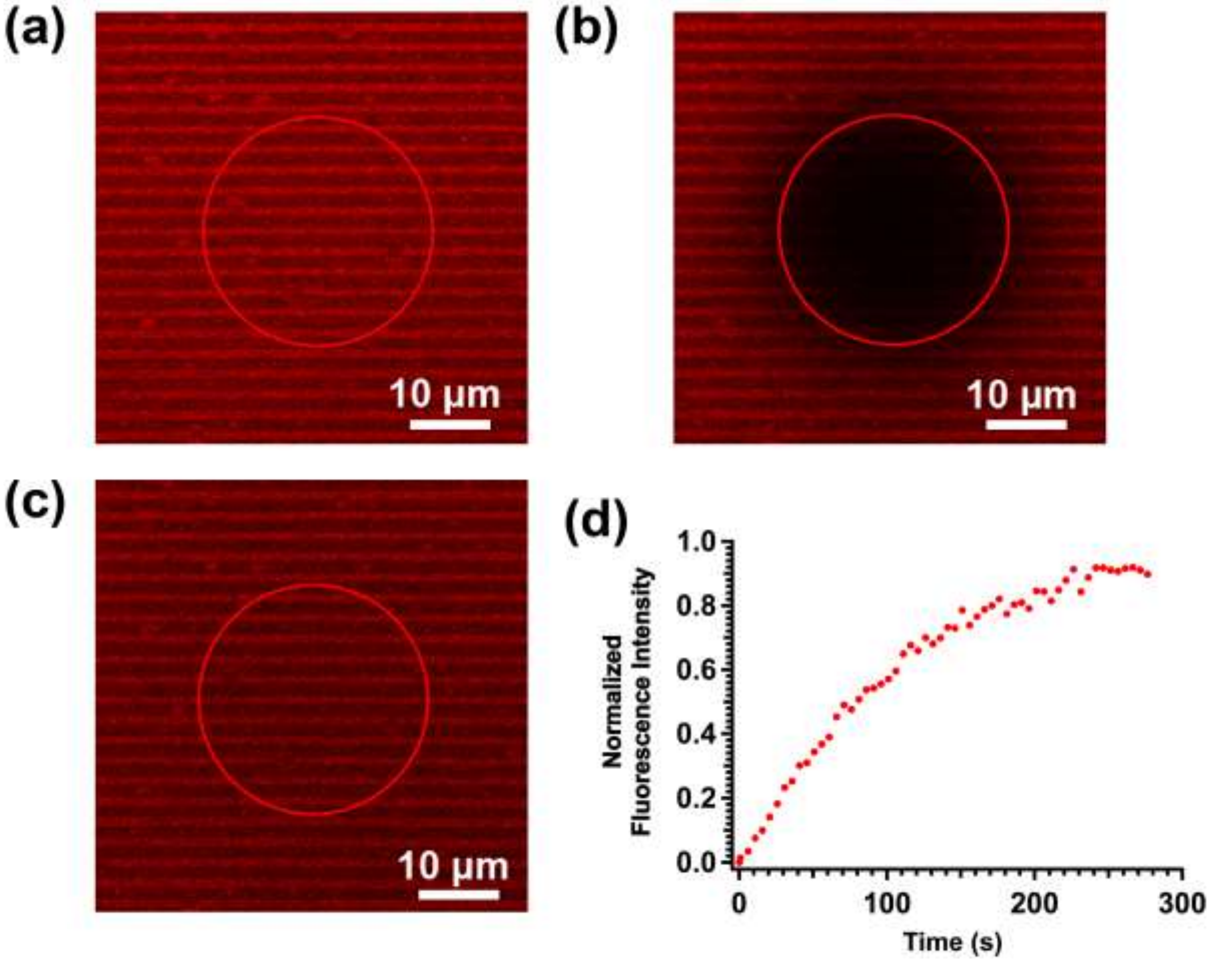

Figure S5: FRAP images of the SLBs on a silicon nanoribbon array before (a), immediately after (b), and 276 seconds (c) after photobleaching, and the corresponding FRAP recovery curve (d).

Capacitance calculations:

In order to compare the capacitance of solution double layer, dielectric layer and the silicon channel, we use the following calculations to roughly estimate their capacitances.

$$` _{Çä} = \frac{\varepsilon_M \varepsilon_{êlë}}{\lambda_a H \mp_p} = \frac{UKUR \times NM^{-NO} \times UM}{MKQPS \times NM^{-V} H MKTQ \times NM^{-V}} = MKSMO \times NM^{-NO} \, c \, L \mu \, ã^O$$

$$` _{çñ} = \frac{\varepsilon_M \varepsilon_{êlçñ}}{í_{çñ}} = \frac{UKUR \, \tilde{} \, NM^{-NO} \, \tilde{} \, V}{OM \times NM^{-V}} = MKMPVU \times NM^{-NO} \, c \, L$$

$$C_{Si} \approx \frac{\varepsilon_{0}\,\varepsilon_{r,si}}{t_{si}} = \frac{8.85\times10^{-12}\times11.7}{40\times10^{-9}} = 0.00259\times10^{-12}\text{F/}\text{ص}2$$

where ε_{M} is the permittivity of free space, $\varepsilon_{\hat{e}l\ddot{e}}$, $\varepsilon_{\hat{e}l\c{c}\tilde{n}}$ and $b_{\hat{e}l\ddot{e}\acute{a}}$ respectively are the relative permittivity of solution, Al_2O_3 layer and active silicon, λ_{a} and $=\lambda_{p}$ respectively are the Debye length and stern layer thickness for 0.5 M ionic strength, and $\acute{\imath}_{\c{c}\tilde{n}}$ and $\acute{\imath}_{\ddot{e}\acute{a}}$ respectively are the thickness of dielectric layer and silicon channel. According to the calculation results, the double layer capacitance is 2 orders of magnitude larger than the nanoribbon capacitance in this case because of the large relative permittivity of solution and the small Debye length at high ionic strengths, and can therefore be neglected from our calculations.

Yield and statistics:

The experiments were performed a total of 10 times. Of these, current spikes were observed 6 times. In 3 experiments, there was a high leakage of the lipid bilayer indicating pinholes or other failures in the formation of a good seal. In one experiment, no obvious spikes or steps were observed. The table summarizes the findings.

Ion channel	Concentration (gA or Al)	Electrolyte	Voltage	Current Spikes or steps	Current Amplitude	Experiment dates
Alamethicin	10 μg/ml	500 mM KCl	100 mV	Spikes	20 to 50 pA	5/26/2015
		500 mM KCl	100 mV	Not good sealing		6/2/2015
Alamethicin	10 μg/ml	500 mM KCl	100 mV	Spikes and steps	5-10 pA<br>~50 pA	7/15/2015
		500 mM KCl	100 mV	Not good sealing		8/6/2015

Alamethicin	1 µg/ml	500 mM KCl	100 mV<br>200 mV	Spikes and steps	50 pA<br>100~300pA	3/21/2016
Alamethicin	2 µg /ml	500 mM KCl	100 mV<br>200 mV	Spikes and steps	50~600 pA	7/14/2016
		500 mM KCl	100 mV	Not good sealing		7/13/2016
Alamethicin	2 µg/ml	500 mM KCl	100 mV	No obvious spikes or steps		8/3/2016
Alamethicin	2 µg/ml	500 mM KCl	100 mV 200 mV	A few of spikes	~50 pA	8/9/2016
Gramicidin	2 µg/ml	500 mM KCl	100 mV	Spikes and steps	5~10 pA	8/4/2015
